# Beneficial effects of non-invasive physical plasma on human periodontal ligament cells *in vitro*

**DOI:** 10.3389/fmed.2024.1443368

**Published:** 2024-11-19

**Authors:** Benedikt Eggers, Lennard Seher, Jana Marciniak, Tristan Pauck, James Deschner, Sigrun Eick, Matthias Bernhard Stope, Franz-Josef Kramer, Erika Calvano Küchler, Christian Kirschneck, Marjan Nokhbehsaim, Svenja Beisel-Memmert

**Affiliations:** ^1^Department of Oral, Maxillofacial and Plastic Surgery, University Hospital Bonn, Bonn, Germany; ^2^Department of Orthodontics, University Hospital Bonn, Bonn, Germany; ^3^Department of Gynecology and Gynecological Oncology, University Hospital Bonn, Bonn, Germany; ^4^Department of Periodontology and Operative Dentistry, University Medical Center of the Johannes Gutenberg University, Mainz, Germany; ^5^Department of Periodontology, School of Dental Medicine, University of Bern, Bern, Switzerland; ^6^Section of Experimental Dento-Maxillo-Facial Medicine, University Hospital Bonn, Bonn, Germany

**Keywords:** human periodontal ligament cells, periodontology, cold plasma, non-invasive physical plasma, non-thermal plasma, antimicrobial

## Abstract

**Introduction:**

Periodontitis is a chronic inflammatory disease of the periodontium that can lead to the loss of affected teeth if left untreated. It is induced by a multifactorial process centered on microbial pathogens such as Fusobacterium nucleatum *(F.n.)*. Non-invasive physical plasma (NIPP), a highly reactive gas, has become a focus of research, not only for its hemostatic, proliferation-enhancing and apoptotic properties, but also for its antimicrobial potential. The objective of this study was to examine the impact of NIPP on human periodontal ligament (PDL) cells that had been induced into a state of periodontal infection in vitro.

**Methods:**

Initially, the solitary effect of NIPP was evaluated by measuring temperature and pH and analyzing reactive oxygen species (ROS). Additionally, DAPI and phalloidin staining were employed to investigate possible cytotoxic effects. The cells were pre-incubated with *F.n*. and treated with NIPP after 24 hours. Interleukin (IL)-6 and IL-8 were analyzed at mRNA and protein levels, respectively, by real-time PCR and ELISA.

**Results:**

NIPP alone had no significant effect on PDL cells. However, the *F.n*.-induced upregulation of IL-6 and IL-8 was counteracted by NIPP.

**Discussion:**

Thus, the utilization of NIPP may be regarded as a promising therapeutic strategy for the treatment of periodontal diseases.

## 1 Introduction

Inflammatory diseases of the tooth-supporting tissue (periodontium) affect−depending on the definition−up to 90% of the world’s population (1, 2). This includes gingivitis, a reversible form of periodontal disease, but also periodontitis, that is characterized by irreversible bone and attachment loss and continues to pose a major challenge to the dental profession (3).

The periodontium is comprised of several components, including gingiva, bone, root cementum, and the periodontal ligament. Collagen fibers, also known as Sharpey fibers, radiate into the root cementum and alveolar bone on all sides, providing an anchor for the tooth root within the socket and bridging the periodontal ligament space. These collagen fibers are formed by PDL cells, which have different phenotypes, such as fibroblast like, osteoblast like and cementoblast like (4, 5). PDL cells are capable of secreting cytokines and chemokines, thereby contributing to the host’s immuno-inflammatory response. Compared to PDL cells from periodontally healthy individuals, inflamed PDL cells show reduced immunomodulatory activity and secrete higher levels of inflammatory cytokines−they also exhibit reduced regenerative potential, such as lower ALP activity (6–8). As a result, these cells influence the course and extent of periodontal diseases, which in the long run leads to the destruction of the tissue (3, 9). However, the rapid degradation of the tissue is primarily mediated by the bacterially induced immunological inflammatory response (10).

In a healthy state, there is an ecological balance between the polymicrobial communities and the host’s immune system. However, various destabilizing factors can destroy the homeostatic balance and lead to a shift in the microbial community towards periodontally destructive species, thus triggering periodontitis (11, 12). The most important bacterial species of periodontitis are periodontal pathogens such as *Porphyromonas gingivalis* (*P.g.*), *Tannerella forsynthia* (*T.f.*), *Tannerella denticola* (*T.d.*) or *Fusobacterium nucleatum* (*F.n.*) (13–15).

Mechanical removal of the supra- and subgingival biofilm, i.e. reduction of the bacterial load, is the fundamental part of periodontal treatment (16). This can also be aided by the surgical procedure in which the pockets are reduced or eliminated by means of regenerative or resective surgery. In addition, the use of local or systemic antibiotics can help eliminate pathogens, although this is becoming problematic in the context of increasing antibiotic resistance (17). All in all, the individual optimization of oral hygiene plays an important role in the possible progression of the disease (18, 19). However, the long-term goal of these methods is to stop the progression of the disease and to stabilize the periodontal conditions.

In recent years, non-invasive physical plasma (NIPP) has emerged as a promising new treatment modality, exhibiting potential to modulate the inflammatory response of human cells (20–22). Starting from the states of matter ‘solid’, ‘liquid’ and ‘gaseous’, which can be transformed into each other by altering the pressure and temperature, NIPP is generated from the gaseous state by adding further energy. A special feature of physical plasma, which has been used in industry for many years, is the temperature, which at 37°C to 40°C is within the range of body temperature, allowing it to be used on patients (22). In the course of its use on patients, a wide range of beneficial effects have been described, including antimicrobial, wound healing and hemostatic properties (23–25). Interestingly, NIPP also appears to have an apoptotic effect on some types of cancer cells (26). However, the precise molecular and cellular mode of action of NIPP is not yet known. During application, various highly reactive oxygen species (ROS) are produced, which are presumably responsible for the biomedical effects (27, 28).

The potential of NIPP, particularly in the oral cavity, has yet to be sufficiently researched. If the results of the above-mentioned studies are confirmed, NIPP could be used for both antibacterial purposes and to promote regeneration without damaging the tissue. Therefore, the rationale of the present study was to find out whether NIPP exerts an anti-inflammatory effect on PDL cells in a microbial environment. In a previous study, we have already shown the effect of NIPP on *F.n.* stimulation in human gingival fibroblasts (29). Nevertheless, the precise extent to which NIPP exerts an effect on the molecular processes of other cells within the periodontium in the presence of *F.n.* remains unknown. Therefore, the aim of the present study was examine the effects of NIPP on the inflammatory response of PDL cells under microbial stimulation, monitored by the classic inflammatory markers interleukin (IL)-6 and IL-8. The objective is to pave the way for future clinical applications of NIPP, which could facilitate the treatment of periodontitis and thus improve oral health.

## 2 Materials and methods

### 2.1 NIPP treatment

The DBD device Plasma One (Plasma MEDICAL SYSTEMS, Nassau, Germany), was used with instrument probe PS30, at intensity level 5 (Supplementary Figures 1A–C).

### 2.2 Determination of temperature, pH, and UV-C

During NIPP treatment, the temperature was measured at various points using the Optris PI 400i infrared camera with Optri PIX Connect analysis software (Optris, Berlin, Germany). The measurement was taken for the Probe tip, discharge, cell culture medium, and counter electrode. In addition, the pH changes of the medium during the NIPP treatment were determined at different time points using a benchtop pH50 VioLab (Carl Roth, Karlsruhe, Germany).

A sensor SUV 20.2 with reference radiometer MUV 2.4WR (both IL Metronic Sensortechnik, Ilmenau, Germany) was used to determine the UV-C emission. The sensor has a sensitivity range of 220–280 nm and is traceably calibrated. For the distance- and angle-dependent measurement of UV-C intensity, the NIPP probe and UV-C detector were installed on tripod stands and brought into the measurement positions using measuring scales (distances: 1 cm, 5 cm, 10 cm; angles: 0°, 90°, 180° in the YZ plane and 0° and 180° in the XY plane).

### 2.3 Measurement of ROS during NIPP treatment

For the O_3_ measurement in the gas phase, the O_3_−specific detector (ZE25-O3 Ozone Sensor, Electronics Technology, Zhengzhou, China) was used with an Arduino UNO r3 microcontroller (Arduino S.r.l., Monza, Italy). The measurement was performed continuously for 200 s at a detector-probe tip distance of 10 cm.

A Quantofix Relax reflectance photometer (Macherey-Nagel, Düren, Germany) with Quantofix peroxide 25 tests (H_2_O_2_; measuring range 0.0–25 mg/L), Quantofix nitrite (NO_3_− measuring range 0.0–80.0 mg/L), and Quantofix nitrate 100 (NO_3_^–^; measuring range 0.0–100.0 mg/L) was employed to determine selected ROS in the aqueous phase.

### 2.4 Cell culture

Human PDL cells (Lonza, Basel, Switzerland) were propagated in 75 cm^2^ flasks (Greiner bio-one, Frickenhausen, Germany) in Dulbecco’s modified essential medium (DMEM; Invitrogen, Waltham, MA, USA), supplemented with 10% fetal bovine serum (FBS; Invitrogen) and 100 units/mL penicillin and 100 μg/mL streptomycin (Invitrogen), and maintained in 37 °C humidified atmosphere with 5% CO_2_. The medium was replaced every 2–3 days. For mRNA and protein analysis, PDL cells were seeded in 35 mm Petri dishes (Greiner bio-one). For the morphological analysis, cells were cultivated on cover slips (Thermo Fisher Scientific, Waltham, MA, USA). One day prior to the experiments FBS concentration in the culture medium was reduced to 1%.

### 2.5 NIPP treatment of PDL cells

PDL cells in the presence or absence of *F.n.* were treated with NIPP for various application times as previously described (29, 30). Untreated PDL cells served as control.

### 2.6 Determination of endogenous ROS generation in PDL cells

The endogenously generated ROS were detected with the CellROX^®^ Deep Red Reagent according to the manufacturer’s instructions. The intracellular cellular ROS production was then visualized after 10 min and 60 min using the ZOE Fluorescent Cell Imager (Bio-Rad, Hercules, CA, USA). Additionally, quantification of ROS production was performed using a fluorescent plate reader at 665 nm (Promega, Madison, WI, USA).

### 2.7 Cell morphology

PDL cells were fixated with 4 % paraformaldehyde (Sigma-Aldrich, Saint-Louis, Missouri, USA) for 10 min and permeabilized in 0.1 % Triton X-100 (Sigma-Aldrich) for 5 min. In order to label actin filaments, cells were incubated for a period of 60 minutes with phalloidin (Sigma-Aldrich). The DNA was stained by incubating DAPI (Sigma-Aldrich) for 5 min. Rinsing with Phosphate Buffered Saline (PBS; Invitrogen, Waltham, MA, USA) was performed between the staining steps. After mounting with Mowiol (Carl-Roth, Karlsruhe, Germany), the cells were analyzed with the ZOE Fluorescent Cell Imager (Bio-Rad).

### 2.8 Inflammation model

To mimic a microbial environment, PDL cells were pre-incubated with lysates of inactivated *F.n.* (ATCC 25586) 24 h prior to NIPP application. The bacteria had been inactivated by suspension in PBS (OD_660_ = 1.0, corresponding to 1.2 × 10^9^ bacterial cells/mL) and sonication (160 W for 15 min, twice).

### 2.9 Analysis of gene expression

RNA was isolated with the RNeasy Mini Kit (Qiagen, Hilden, Germany) according to the manufacturer’s information. Next, 1 μg of total RNA was transcribed into cDNA using the iScript Select cDNA Synthesis Kit (Bio-Rad Laboratories, Munich, Germany). One μl of cDNA, 2.5 μl of commercially available primers Glyceraldehyde-3-phosphate dehydrogenase (GAPDH), IL-6, IL-8 (QuantiTect Primer Assay, Qiagen), 9 μl of deionized water, and 12.5 μl of SSoAdvanced Universal SYBR Green Supermix (Bio-Rad) was incubated in the iCycler iQ5 detection system (Bio-Rad). The following amplification protocol was used: 95 °C for 5 min, followed by 40 cycles of denaturation at 95 °C for 10 s and combined annealing/extension at 60 °C for 30 s. Data was analyzed using the comparative threshold cycle method.

### 2.10 Analysis of protein levels

The IL-6 and IL-8 protein levels in cell culture supernatants were analyzed by specific enzyme-linked immunoassay (ELISA) Kits (Bio-Techne, Minneapolis, MN, USA) according to the manufacturer’s instructions. A microplate reader (Epoch™ Microplate Spectrophotometer, BioTek Instruments, Winooski, VT, USA) at 450 nm was employed to read out the immunoassays. The protein concentrations of IL-6 and IL-8 were normalized to the total protein concentration by the Pierce BCA Protein Assay Kit (23227, Thermo Scientific, Pierce Biotechnology, Rockford, IL, USA) and a microplate reader at 570 nm.

### 2.11 Statistical analysis

Statistics was done with GraphPad Prism version 10 software (GraphPad Software, Inc., La Jolla, CA, USA). Nonparametric tests (Kruskal–Wallis test with post hoc Dunn’s multiple comparisons test) and parametric tests (One-way ANOVA with post hoc Tukey multiple comparisons test) were applied. The observed differences were considered statistically significant, with *p*-values below 0.05.

## 3 Results

### 3.1 Basic NIPP characterization I: influence of NIPP on temperature, pH and ROS in cell culture medium

**FIGURE 1:**
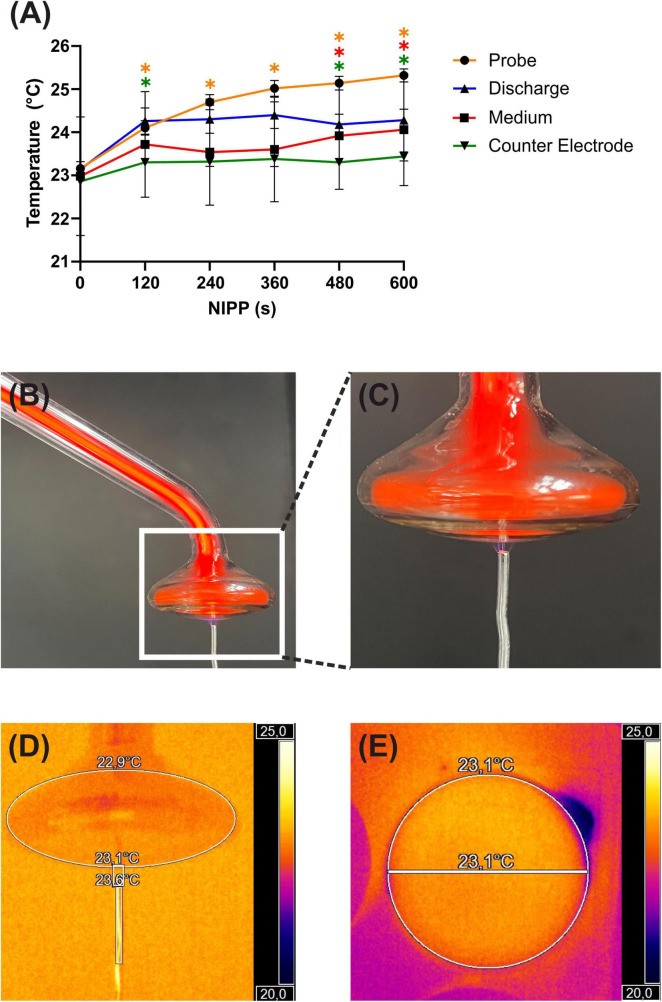
Basic characterization of NIPP during treatment: **(A)** temperature of the NIPP device and treated objects, *n* = 5. * Statistical significance compared to NIPP 0 s (*p* < 0.05). **(B)** Plasma ONE with probe PS30 and counter electrode in discharge mode. **(C)** Magnification of the probe tip. **(D)** Thermal image of the probe tip using the Optris PI 400i infrared camera with Optri PIX Connect analysis software (Optris, Berlin, Germany). **(E)** Thermal image of the bottom view of the probe tip.

First, the main physico-chemical characteristics of the NIPP device (temperature changes at the probe, discharge zone, counter electrode and treated media surface) were studied (Figures 1A–E). The temperature of the probe increased continuously with increasing treatment time: from 23.2°C (0 s) to 24.7°C (240 s) and up to 25.3°C (600 s). The temperature of the effluent did not change with increasing treatment time and averaged 24.1°C. The temperature of the media surface increased slightly over 600 s from 23.0°C to 24.1°C. However, there was no significant temperature change within the first 120 s. The direct discharge on the counter electrode showed a slight temperature change from 22.9°C to 23.3°C. Overall, the temperature in the culture medium and in the NIPP device did not increase during operation to the extent that thermal effects on the cells would be expected.

**FIGURE 2:**
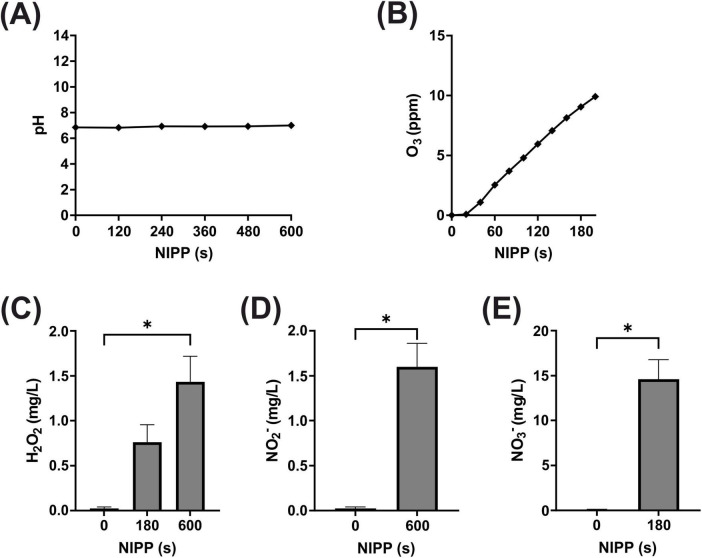
Basic characterization of NIPP during treatment of medium: **(A)** pH of medium, *n* = 5 **(B)** O_3_ concentration in medium, *n* = 2 **(C)** H_2_O_2_ concentration in medium, *n* = 6 **(D)** NO_3_^–^ concentration in medium, *n* = 5 **(E)** NO_2_^–^ concentration in medium, *n* = 5. *Statistical significance compared to control (*p* < 0.05).

Next, we analyzed the pH of the medium during NIPP treatment. As shown in Figure 2A there was no change in medium pH during 600 s of NIPP treatment. Another important parameter, particularly regarding patient and user protection, was the UV-C emission of the device. It was found that UV radiation between 200 and 280 nm could not be detected at any distance greater than 1 cm from the tip of the probe and at any angle to the probe (data not shown).

The generation of high energy NIPP and its subsequent interaction with the ambient air produces a large number of ROS. These have been shown to be significantly involved in the biomedical effect of NIPP and were therefore also analyzed. In the gaseous phase around the glass probe, the O_3_ concentration increased and reached 3.52 ppm after 60 s and 8.04 ppm after 120 s (Figure 2B). Since ROS are also water-soluble, the comparatively long-lived ROS H_2_O_2_, NO_2_^–^ and NO_3_^–^ were detected in the aqueous phase of the cell culture medium after NIPP treatment. Due to the low sensitivity of the measuring system, longer NIPP treatment times of 60, 180, and 600 s were applied in these experiments. While no H_2_O_2_ was detectable in the medium after 60 s, the H_2_O_2_ concentration subsequently increased to 0.8 mg/L (180 s) and 1.4 mg/L (600 s; Figure 2C). NO_2_^–^ was only measurable after 600 s (1.6 mg/L; Figure 2D). NO_3_^–^ could be detected after 180 s of NIPP treatment (14.6 mg/L; Figure 2E). After 600 s, however, no further increase in concentration was observable, as NO_2_^–^ that then also appeared interfered with the NO_3_^–^ detection reaction. Overall, however, ROS analyses confirmed that the long-lived ROS marker molecules O_3_, H_2_O_2_, NO_2_^–^ and NO_3_^–^ were detectable after NIPP treatment of the cell culture medium.

### 3.2 Basic NIPP characterization II: impact of NIPP on the production of ROS in PDL cells

**FIGURE 3:**
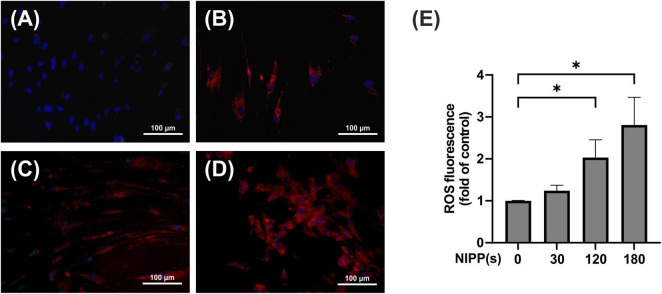
Immunofluorescence staining of ROS production in PDL cells, 10 min after NIPP treatment. Cells were treated for 0 s **(A)**, 30 s **(B)**, 120 s **(C)**, 180 s **(D)**. The scale bar represents 100 μm. ROS fluorescence was quantified using a fluorescent reader **(E)**. *n* = 4. *Statistical significance (*p* < 0.05).

The next step was to test whether treating PDL cells with NIPP generates intracellular ROS. The CellROX Deep Red reagent is a membrane-permeable low-molecular compound that can be used to label living cells. The endogenous formation of ROS leads to the oxidation of the intracellular reagent. As a result, the reagent can no longer permeate the cytoplasmic membrane and leave the cell, and the molecule exhibits a strong, highly detectable and well quantifiable fluorescence signal. The live cell imaging experiments revealed that 10 min after NIPP treatment there was a clear and dose-dependent generation of endogenous ROS, which was statistically significant after 120 and 180 s of treatment (Figures 3A–D). In contrast, no ROS-induced fluorescence signals were detectable 60 min after NIPP treatment (data not shown).

### 3.3 Effect of NIPP on PDL cell morphology and viability

**FIGURE 4:**
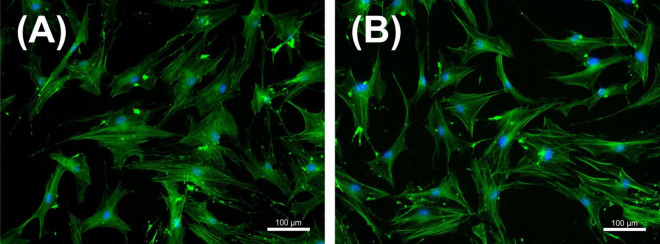
Immunofluorescence staining of periodontal ligament (PDL) cells with DAPI (blue staining of the nucleus) and phalloidin (green staining of the cytoskeleton) that were left untreated **(A)** or treated with NIPP for 30 s at 24 h **(B)**. NIPP treatment showed no cytotoxic effects. The scale bar represents 100 μm.

Following NIPP characterization, we focused on an application time of 30 s, in accordance with our previous study (29). Our first objective was to observe whether the selected NIPP treatment time had a negative effect on viability and cell morphology of PDL cells. Phalloidin-DAPI double staining revealed an intact cytoskeleton and undamaged nucleus 24 h after NIPP treatment (Figures 4A, B).

### 3.4 Influence of *F.n*. on IL-6 and IL-8 regulation in PDL cells

**FIGURE 5:**
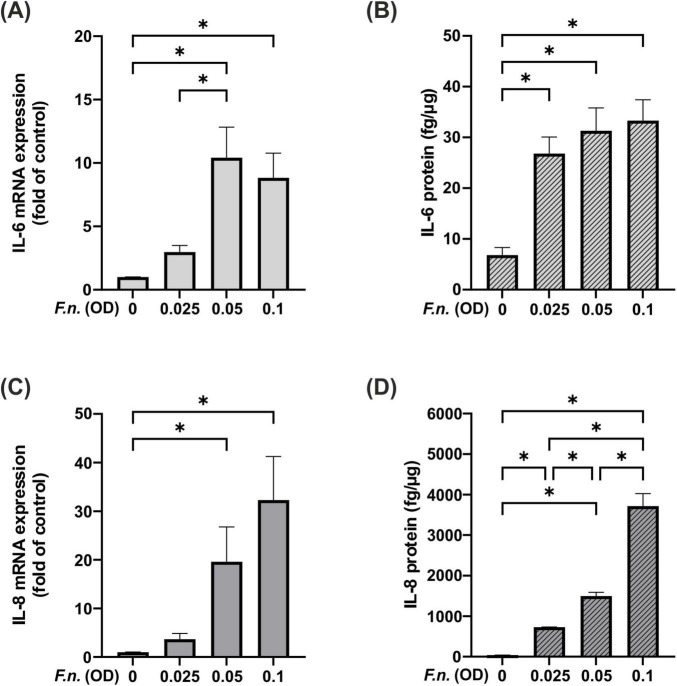
IL-6 and IL-8 levels in PDL cells after pre-incubation with inactivated *F.n.* at different concentrations (OD = 0.025, 0.05, 0.01). **(A)** IL-6 mRNA expression at 24 h, n = 9 **(B)** IL-6 protein level at 48 h, *n* = 12 **(C)** IL-8 mRNA expression at 24 h, *n* = 9 **(D)** IL-8 protein level at 48 h, *n* = 12. *Statistical significance (*p* < 0.05).

Having studied the effects of NIPP on PDL cells under control conditions, PDL cells were exposed to *F.n.* to mimic a microbial environment. To this end, PDL cells were incubated with the periodonto-pathogen in different concentrations. A dose-dependent upregulation of IL-6 was observed at mRNA level after 24 h, which was also reflected at protein level after 48 h (Figures 5A, B). A similar effect was observed for the expression of IL-8 at mRNA level after 24 h and at protein level after 48 h (Figures 5C, D). As all *F.n.* concentrations resulted in a dose-dependent upregulation at protein level, we decided to use the lowest *F.n.* concentration (OD: 0.025) for subsequent experiments.

### 3.5 Influence of NIPP on IL-6 regulation in *F.n*. pre-incubated PDL cells

**FIGURE 6:**
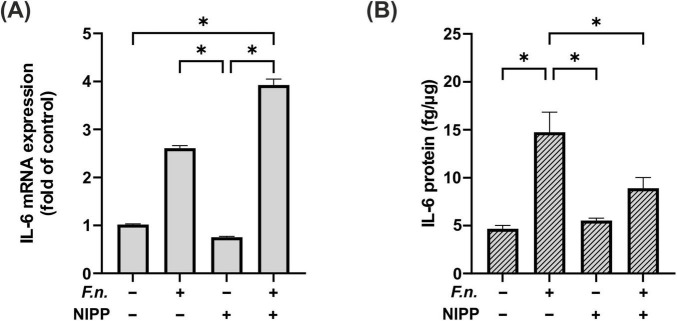
IL-6 regulation in PDL cells after 24 h pre-incubation with inactivated *F.n.* at OD 0.025 (+) and after 30 s of NIPP treatment (+). **(A)** IL-6 mRNA expression at 24 h, *n* = 6 **(B)** IL-6 protein level at 48 h, *n* = 12. * Statistical significance (*p* < 0.05).

As a next step the impact of NIPP on *F.n.* pre-incubated PDL cells was investigated. NIPP did not directly affect IL-6 regulation at mRNA level (Figure 6A). There was a significant increase in IL-6 expression following NIPP treatment of *F.n.* pre-incubated PDL cells as compared to control. However, the IL-6 expression did not change significantly as compared to treatment with *F.n.* alone. Interestingly, at protein level, NIPP treatment counteracted the upregulation of IL-6 due to *F.n.* pre-incubation (Figure 6B).

### 3.6 Influence of NIPP on IL-8 regulation in *F.n*. pre-incubated PDL cells

**FIGURE 7:**
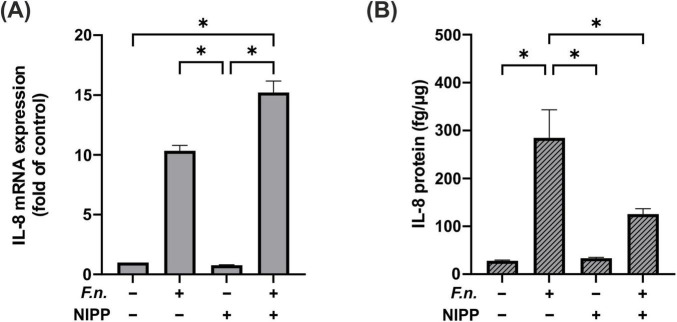
IL-8 regulation in PDL cells after 24 h pre-incubation with inactivated *F.n. at OD 0.025* (+) and after 30 s of NIPP treatment (+). **(A)** IL-8 mRNA expression at 24 h, *n* = 6 **(B)** IL-8 protein level at 48 h, *n* = 12. * Statistical significance (*p* < 0.05).

Similarly, we examined the effect of NIPP on IL-8 in PDL cells after *F.n*. exposure. We observed a significant increase in IL-8 gene expression in cells, which were subjected to the combination of *F.n*. and NIPP (Figure 7A). However, this significant increase was not observed at the protein level. In parallel to IL-6 levels, the elevation of IL-8 by *F.n.* was counteracted by NIPP treatment (Figure 7B).

## 4 Discussion

Our data demonstrate for the first time that NIPP treatment may counteract bacterial induced proinflammatory oral cell response during the course of an infection. Applying an infection model in which PDL cells were incubated with *F. n.* lysates, a single short-term treatment of 30 to 120 s with NIPP already led to a significant reduction of inflammatory effects.

The innovative and inexpensive physical treatment method with NIPP is characterized by the fact that it is gentle on surrounding cells and tissue, is almost painless and no undesirable therapeutic effects have been described to date. This could also be indicated *in vitro*. Neither the surface temperature of the treated samples nor the pH of the cell culture medium changed as a result of the NIPP treatment. The cell morphology also did not allow any conclusions to be drawn about cytotoxic changes in the PDL cells.

Furthermore, it was demonstrated that NIPP also led to the generation of biomedically effective ROS also in the context of a peridontitis therapy approach. O_3_ was detected in the gaseous phase, while NO_2_^–^, NO_3_^–^ and H_2_O_2_ accumulated in the aqueous phase. According to the current state of knowledge, ROS in general, but in particular the above-mentioned and comparatively stable ROS, are the main factors in antimicrobial including antiinflammatory NIPP efficacy (22, 29, 31).

An infectious cell environment was mimicked by stimulating PDL cells with *F. n.* lysates. This led to the induction of pro-inflammatory IL-6 and IL-8 indicating an inflammatory cell response of the *in vitro* cell culture model. Subsequent treatment with NIPP was able to reduce this interleukin expression again, which suggests an anti-inflammatory therapeutic effect of the procedure. Interestingly, however, stimulation of *F. n.* preincubated cells showed an induction of IL-6 and IL-8 at the mRNA level and a decrease at the protein level. Similar effects were also observed previously in gingival fibroblasts (29). Even if the clinical effects of NIPP are reflected only in protein levels and not in mRNA expression, the opposite regulation seen in both PDL cells and gingival fibroblasts may indicate a specific mechanism regulated by NIPP. This could be due to posttranslational regulatory mechanisms that may be regulated by NIPP (32). Furthermore its possible, that degrading enzymes activated by ROS directly or indirectly which than could interfere with the cytokines induced (33). However, further studies should clarify this discrepancy between the mRNA and protein levels. Regarding the anti-inflammatory effect of NIPP, similar effects have been demonstrated in fibroblast-like synoviocytes from patients with rheumatoid arthritis. Also here, NIPP treatment led to a reduction in inflammatory factors, such as NF-κB or IL-6 (34). NIPP has also been shown to reduce inflammation and improve wound healing in diabetic wounds (35).

Interestingly, another study has shown that NIPP treatment promotes the induction of IL-6 and IL-8 in skin cancer cells (36). However, it is known that NIPP can have different or even opposite effects on the cell viability of physiologically healthy and malignant cells. NIPP increases metabolic activity, migration and proliferation in benign cells (37, 38), but has opposite effects on malignant cells, causing them to undergo apoptosis (39, 40). The stimulatory effects of NIPP on cell viability in PDL cells have already been shown in our previous study (30). It appears that skin cancer cells and benign oral cells also respond differently to NIPP therapy in terms of IL-6 and IL-8 secretion. However, further studies are needed to investigate the background of the different reaction patterns.

We have focused on the cytokines IL-6 and IL-8 as being the key drivers of inflammatory processes in periodontitis (41, 42). The immunoregulatory cytokine IL-6 is secreted by immune and tissue cells. It acts as a chemoattractant for lymphocytes and controls the differentiation of B and T cells (43–45). Consequently, IL-6 is involved in inflammatory immune diseases such as Crohn’s disease (46), systemic lupus erythematosus (47), and also periodontitis (48). It is therefore considered a diagnostic factor for periodontitis and is seen in patients with an increased alveolar bone loss and an overrepresentation of periodontal pathogens (49, 50).

IL-8 has pro-inflammatory effects and is responsible for the activation and migration of neutrophils into tissue (51). It is released in the affected tissue in various inflammatory diseases (52), including atherosclerosis (53), intestinal inflammation (54), and periodontitis (55). For this reason, it is a biomarker employed in the diagnosis of periodontitis (56).

We utilized *F.n.* lysate to experimentally induce a cellular inflammatory response. *F.n.* is an important bridging bacterium that serves as a link between early and late colonizers in oral biofilms (13). It plays an important role in both, gingivitis and periodontitis (57). As demonstrated by other authors, stimulation with *F.n. in vitro* results in the release of proinflammatory cytokines (58–60). However, other bacteria may also be used to induce experimental periodontitis *in vitro:* Yumoto et al. have shown, that *Eikenella corrodonens* stimulates the secretion of TNF-α, IL-6 and IL-8 (61). Similar effects have also been described by stimulation of gingival fibroblasts with *P.g.* (62). However, this study focused on pre-incubation with *F.n.* as this study is intended to complement our previous experiments on gingival fibroblasts (29). The restriction to *F. n.* as bacterial inducer of inflammatory processes in the *in vitro* PDL cell model is therefore a limitation of the present study. Other periodontal pathogens, such as *T.d.*, *P.g.*, and *Prevotella intermedia* should be the focus of future projects. Moreover, it would also be interesting to investigate NIPP effects on the expression rates and activities of further inflammatory mediators such as IL-1β or TNFα.

Moreover, we have used a dielectric barrier discharge (DBD) device to generate NIPP, which, as with all NIPP device types, is generated between two electrodes. In this NIPP device, one electrode with an isolating layer forms the discharge gap (63). The object to be treated forms the second electrode, such as the PDL cells in this study. The device must be in close contact with the periodontal cells to generate NIPP (64). This may be challenging in some clinical situations. However, a clinical application of DBD devices in dentistry has been described for palatal wound healing (65) and antifungal therapy (66). There are other NIPP technologies that do not require close contact with the surface being treated. NIPP jet technology, for example, generates a NIPP effluent similar to a gas flow, which can also be used to reach and treat uneven surfaces. A limitation of the jet technology, however, is that the target area for treatment is limited. Nevertheless, its clinical use in dentistry has been described in the literature: For instance, the application of a NIPP jet has been shown to improve wound healing after gingivectomy (67).

Comparison of *in vitro* NIPP studies in which different NIPP devices from different suppliers were used is only possible to a very restricted extent. The physicochemical properties of the generated NIPP are highly dependent on the technology used and are additionally modulated by other technical variables, e.g. carrier gas and flow rate as well as device parameters such as power and frequency. All these factors have an influence on the composition of the ROS mixture, the concentrations of the individual ROS and thus on the biomedical effect (68, 69). Nevertheless, a comparison can be made between the present results and those of previous studies. These findings indicate that a treatment duration of 30 seconds exerts an anti-inflammatory effect on the cells (29). In previous studies, it was also observed that NIPP effects on wound healing became visible after approximately 60 s (30, 31). It is hypothesized that when NIPP is applied to PDL cells, an antimicrobial effect can be observed initially, followed by a wound healing-promoting effect with increasing application time. This hypothesis requires further investigation.

This study has other limitations: It should be considered that an inactivated *F.n.* was used to induce periodontitis in our experiments. As not only *F.n.* but a lot of other microorganisms play a crucial role in periodontitis (70), these should also be investigated in further studies. It should also be noted that a lysate has been used in our experiments. Further experiments could also be conducted with living bacteria, which exert varying effects on cells depending on their specific virulence factors, such as lipopolysaccharides, proteolytic enzymes, and toxins, when present alone or in whole biofilms. In addition, only PDL cells were examined in this study. As periodontitis affects the entire periodontium and leads to the degradation of hard tissue (71), human osteoblasts, cementoblasts or gingival keratinocytes should also be included in future studies. Moreover, it should be considered that in this study we only treated the cells with a single NIPP application time of 30 s. It is possible that repeated or extended application may have a stronger anti-inflammatory effect. Although we were guided by previous findings that NIPP therapy had a positive effect on growth-regulatory processes in PDL cells (30), longer application times should be further investigated in future studies. Moreover, the present study was conducted with a specific focus on the pro-inflammatory cytokines IL-6 and IL-8. Additional research into anti-inflammatory cytokines and their receptors would be advantageous in order to substantiate the anti-inflammatory effects of NIPP. In addition, it must be considered that the study was an *in vitro* study that can only be applied to the clinical situation to a limited extent. In patients, a number of factors, including age, gender, medication, and living conditions, as well as the degree and extent of existing periodontal disease, may influence the efficacy of NIPP. Our results, however, suggest a potential mechanism of NIPP in clinical practice, and a clinical trial may provide further evidence of its clinical effectiveness.

However, the results of the study could be extrapolated to the clinical situation. Thus, a clinical trial should be conducted to determine whether NIPP has a role in the treatment of periodontitis. As clinical studies in dermatology suggest that NIPP both promotes wound healing (23) and has antibacterial effects (72), it seems reasonable to assume, that NIPP might also have beneficial effects on diseases in periodontal tissues. A single clinical study has also shown that NIPP treatment during periodontal therapy reduces recolonization with periodontopathogens (73). However, further clinical studies are necessary to examine the potential of NIPP in clinical periodontology. Should these anti-inflammatory properties for periodontal disease be validated in clinical trials, NIPP could potentially assist in reducing the prevalence of periodontitis and improving overall oral health.

## Data Availability

The raw data supporting the conclusions of this article will be made available by the authors, without undue reservation.
