# Supplementary figures and images for: Beneficial effects of non-invasive physical plasma on human periodontal ligament cells in vitro
Source: Front Med (Lausanne). 2024 Nov 19;11:1443368. doi: 10.3389/fmed.2024.1443368 (PMC11611554; doi:10.3389/fmed.2024.1443368)

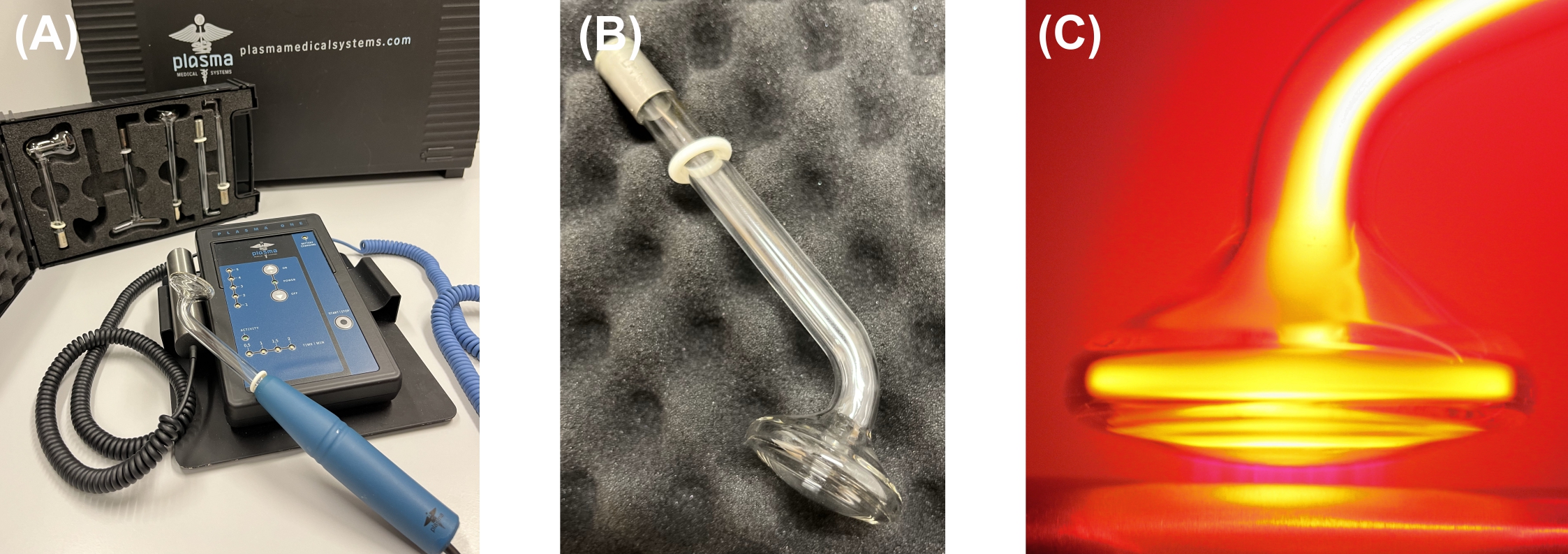

Supplement: Supplementary file 1 [file Image_1.JPEG]
